# Differential incorporation of SUN-domain proteins into LINC complexes is coupled to gene expression

**DOI:** 10.1371/journal.pone.0197621

**Published:** 2018-05-29

**Authors:** Christopher K. May, Christopher W. Carroll

**Affiliations:** Dept. Of Cell Biology, Yale School of Medicine, New Haven, CT, United States of America; Cedars-Sinai Medical Center, UNITED STATES

## Abstract

LInkers of Nucleoskeleton and Cytoskeleton (LINC) complexes, composed of SUN and KASH-domain proteins, span the nuclear envelope and physically connect the nuclear interior to cytoskeletal elements. Most human cells contain two SUN proteins, Sun1 and Sun2, and several KASH-proteins suggesting that multiple functionally distinct LINC complexes co-exist in the nuclear envelope. We show here, however, that while Sun1 and Sun2 in HeLa cells are each able to bind KASH-domains, Sun1 is more efficiently incorporated into LINC complexes under normal growth conditions. Furthermore, the balance of Sun1 and Sun2 incorporated into LINC complexes is cell type-specific and is correlated with SRF/Mkl1-dependent gene expression. In addition, we found that Sun1 has a LINC complex-independent role in transcriptional control, possibly by regulating the SRF/Mkl1 pathway. Together, these data reveal novel insights into the mechanisms of LINC complex regulation and demonstrate that Sun1 modulates gene expression independently of its incorporation into LINC complexes.

## Introduction

A defining feature of eukaryotic cells is the compartmentalization of the genome into a membrane-enclosed nucleus. This separation necessitates that cells communicate information about their environment to the genome across the nuclear envelope. Nuclear pore complexes facilitate chemical signaling to the genome by facilitating the exchange of large (> ~40kDa) macromolecules between the cytoplasm and nucleus [1]. In addition, LInkers of Nucleoskeleton and Cytoskeleton (LINC) complexes propagate mechanical forces across the nuclear envelope to convey information about the extracellular environment to the nuclear interior [2–4]. Mechanical signaling through LINC complexes is critical for cell migration and differentiation [5–7], and disruption of this process has also been linked to a number of pathological conditions, including muscular dystrophies and cancer [8].

LINC complexes are composed of Sad1, UNC84 (SUN)-domain proteins and Klarsicht, ANC-1, Syne Homology (KASH)-domain proteins. KASH-domain proteins extend from the outer nuclear envelope into the cytoplasm and interact with cytoskeletal elements. SUN-domain proteins extend into the nucleoplasm from the inner nuclear membrane (INM) and bind to the nuclear lamina, chromatin, and other INM proteins [9–13]. LINC complexes are formed through the interaction of SUN and KASH-domains in the nuclear envelope lumen, establishing a direct molecular bridge between the cytoskeleton and the nuclear interior [11].

Most vertebrate cell types express two SUN-domain proteins, called Sun1 and Sun2, and several related KASH domain-containing Nesprin proteins [10, 14]. Biochemical studies indicate that SUN-domains and KASH-domains interact promiscuously [15–17]. Thus, multiple different LINC complex forms could co-exist within the nuclear envelope of a given cell type [18]. LINC complexes have been implicated in cytoskeletal dynamics and organization during cellular processes such as spreading, or migration [4, 16, 19–25], and early studies indicated that SUN-domain and KASH-domain proteins played largely redundant roles during development [26–29]. More recent studies, however, demonstrated that related LINC complex proteins play different, or even opposing roles [25, 30, 31]. A fundamental challenge is to uncover the mechanisms that control the abundance of different LINC complex forms to establish the functional capacity of the nuclear envelope. In addition, relatively little is known about whether LINC complex-independent functions of SUN and KASH proteins contribute to their functions.

We recently demonstrated that Sun1 inhibits while Sun2 promotes activation of a positive feedback loop comprised of the small GTPase RhoA and the Serum Response Factor/Megakaryoblastic Leukemia 1 (SRF/Mkl1) transcription factor/co-activator complex in HeLa cells [31]. In this paper, we investigated the biochemical basis for the opposing roles of Sun1 and Sun2 in this signaling system. Our data show that in HeLa cells, in which the inhibitory function of Sun1 is dominant, LINC complexes are biased towards Sun1 inclusion. Ectopic activation of SRF/Mkl1-dependent gene expression, which flips HeLa cells into a state favoring Sun2 LINC complex function, triggered a specific increase in Sun2 LINC complex abundance. Interestingly, overexpression of Sun1 that was unable to form LINC complexes was sufficient to reduce SRF/Mkl1 target gene expression. Together, these data suggest that Sun1 inhibits signaling through LINC complex-independent inhibition of Mkl1/SRF gene expression in the nucleus.

## Results and discussion

### Sun1 is more efficiently incorporated into LINC complexes than Sun2

**Fig 1:**
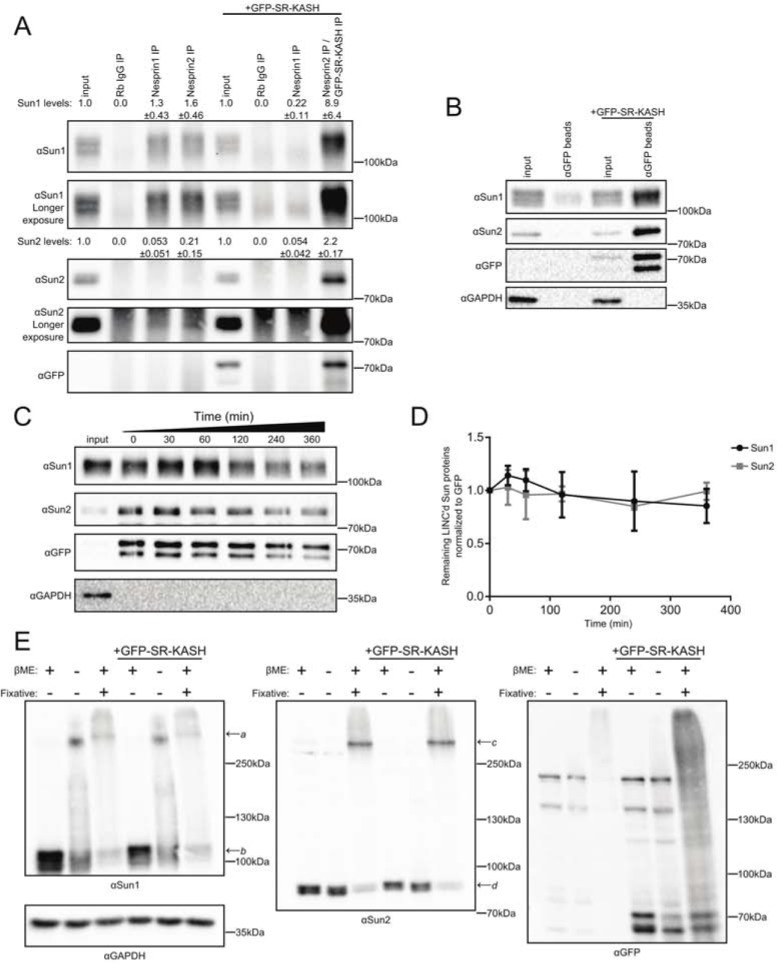
Sun1 is preferentially incorporated into LINC complexes over Sun2. (A) Endogenous Nesprin1 and Nesprin2 immunoprecipitations of SUN proteins. GFP-SR-KASH outcompetes Nesprin1 binding but is recognized by Nesprin2 immunoprecipitation. Immunoprecipitation quantifications were conducted by subtracting the nonspecific Rb IgG lane and normalizing to respective input levels (mean±SEM, n = 3). The specificity of the Sun1 and Sun2 antibodies used in these and all subsequent studies was verified using siRNAs specific for each gene (Part A in S1 Fig). (B) Representative blots of GFP-SR-KASH overexpression and pulldown with αGFP beads. (C) Purified LINC complex off-rate experiment shows stable binding for both Sun1 and Sun2 *in vitro* over several hours. (D) Quantification of blots shown in C normalized to 0min time point and GFP (mean±SEM, n = 3). (E) Sun1 oligomers (*a* ~305kDa) and Sun2 oligomers (*c* ~275kDa) are preserved in nonreducing (βME-), or fixative with reducing conditions. Further, Sun1, but not Sun2, oligomers are disulfide stabilized. In reducing conditions without fixative, only monomers of Sun1 (*b* ~110kDa) and Sun2 (*d* ~82kDa) are present. This distribution is insensitive to GFP-SR-KASH overexpression. GAPDH is a loading control.

To investigate the biochemical basis for the opposing functions of Sun1 and Sun2 in HeLa cells, we immunoprecipitated their Nesprin binding partners from whole cell extracts. Sun1 consistently co-precipitated with both Nesprin1 and Nesprin2 (Fig 1A). Surprisingly, however, we found that Sun2 was almost undetectable in Nesprin immunoprecipitates (Fig 1A). Nesprin1 and Nesprin2 could not be detected by western blot in these experiments, likely due to their large size, multiple isoforms, and/or relatively low abundance in HeLa cells [32, 33]. In order to demonstrate the specificity of the nesprin antibodies, we expressed GFP-SR-KASH, which contains the KASH-domain, transmembrane segment, and membrane-proximal cytoplasmic sequences derived from Nesprin2. Consistent with previous reports demonstrating that GFP-SR-KASH efficiently blocks LINC complex assembly by outcompeting endogenous Nesprin proteins for SUN-domain binding, GFP-SR-KASH expression inhibited the co-precipitation of Sun1 with Nesprin1 (Fig 1A) [30, 31]. Conversely, the Nesprin2 antibody used in this study recognizes the cytoplasmic segment present in the GFP-SR-KASH protein. As a result, increased levels of Sun1 were present in Nesprin2 immunoprecipitates from cells expressing GFP-SR-KASH (Fig 1A). Together, these data demonstrate that the presence of Sun1 in Nesprin immunoprecipitation reactions reflects specific binding to KASH-domain and suggests that Sun1, but not Sun2, is incorporated into endogenous LINC complexes in HeLa cells.

Sun2 did not associate with Nesprin1 or Nesprin2 in cell extracts from unperturbed HeLa cells. A substantial fraction of Sun2, however, was present in Nesprin2 but not Nesprin1 immunoprecipitates after GFP-SR-KASH expression (Fig 1A), suggesting that Sun2 does bind efficiently to the GFP-SR-KASH protein. To test this possibility directly, we precipitated GFP from cells expressing GFP-SR-KASH. We found that both Sun1 and Sun2 efficiently bound to the GFP-SR-KASH protein (Fig 1B). Thus, the failure of Sun2 to bind to endogenous Nesprin proteins does not reflect an intrinsic defect in the ability of Sun2 in HeLa cells to bind to KASH domains.

One explanation for these data is that Sun2 LINC complexes are inherently less stable than Sun1 LINC complexes and fall apart during the immunoprecipitation procedure. To investigate this possibility more quantitatively, we measured the dissociation-rate of Sun1 and Sun2 from the GFP-SR-KASH protein. We found that little to no Sun1 or Sun2 dissociated from the GFP-SR-KASH protein over a six hour time course (Fig 1C and 1D), suggesting that LINC complexes are biochemically stable. LINC complex dissociation is therefore not likely to explain the lack of Sun2 present in Nesprin immunoprecipitations.

As an alternative approach to interrogate SUN-domain protein incorporation into LINC complexes, we measured the levels of free, or unLINC’d, Sun1 and Sun2 in HeLa cells. A GST-KASH peptide from Nesprin2 was immobilized on beads and used to isolate SUN-domain proteins from cell extracts. We found that Sun2 efficiently bound to recombinant GST-KASH but that Sun1 did not, suggesting that most Sun1 in HeLa cells is already stably bound to KASH-peptides (Part A in S1 Fig). To verify that this approach does distinguish free SUN-domain proteins from those already incorporated into LINC complexes, we prepared cell extracts from HeLa cells expressing GFP-SR-KASH protein. We found that exposure of Sun2 to the GFP-SR-KASH protein prior to preparing the cell extract efficiently blocked Sun2 binding the GST-KASH protein (Part B in S1 Fig). No interaction between Sun2 and a mutant GST-KASH protein (GST-KASH^mut^) that lacks the C-terminal 4 amino acids necessary for LINC formation was observed [9, 15], demonstrating the specificity of binding in these experiments.

Structural studies of a KASH-peptide bound to Sun2 indicate that SUN-domain trimerization is a prerequisite for LINC complex assembly [15, 34]. We therefore assessed the oligomerization state of Sun1 and Sun2 in HeLa cells. Previous results have indicated that Sun1 oligomers, potentially in dimers or trimers, were stabilized by disulfide bonds [15, 35]. Consistent with this conclusion, we found that a significant pool of endogenous Sun1 formed disulfide-stabilized species that migrated at the expected molecular weight of a trimer in non-reducing SDS-PAGE gels (Fig 1E). In contrast, Sun2, which lacks luminal cysteine residues outside of the SUN-domain, was monomeric under the same conditions. Oligomers of Sun2 migrating at the approximate size expected for trimers were detected, however, when cells were crosslinked with fixative prior to lysis (Fig 1E). Similar assemblies were also observed for Sun1 after formaldehyde crosslinking. Overexpression of the GFP-SR-KASH protein did not change the migration pattern of SUN-domain proteins regardless of the experimental conditions used. The inability to oligomerize therefore does not explain the failure of Sun2 to form LINC complexes in cells.

When taken together, the data show that Sun1 is more efficiently incorporated into LINC complexes than Sun2 in HeLa cells. These data could reflect differences in their intrinsic biochemical properties. Previous measurements of Sun1 and Sun2 binding to KASH peptide suggest that they have similar affinities with Sun2 being slightly weaker [36]. Our preliminary biochemical data using *in vitro* translated luminal domains from Sun1 and Sun2 also suggest that their affinity for the GST-KASH peptide is comparable (Part C in S1 Fig). These findings are not inconsistent with previous results showing that both Sun1 and Sun2 contribute to Nesprin2 localization in the nuclear envelope [11]. Indeed, we demonstrate below that Nesprin2 partitions to Sun2 in cells in which Sun1 LINC complex assembly is specifically blocked. Instead, our data support the idea that mechanisms exist to control LINC complex assembly in cells. Further, we suggest that Sun1 inhibits the ability of Sun2 LINC complexes to promote SRF/Mkl1 activity by sequestering KASH-domain proteins away from Sun2 [31].

### SRF/Mkl1 signaling promotes Sun2 LINC formation

Our previous data indicated that SUN-domain proteins have opposing roles in regulating signaling through the RhoA-SRF/Mkl1 axis. Specifically, Sun1 inhibited SRF/Mkl1 activity while Sun2 LINC complexes promoted SRF/Mkl1-dependent gene expression in a mechanism that involved regulation of the small GTPase RhoA [31]. In HeLa cells in normal growth conditions, Sun1 is dominant. However, expression of a constitutively active form of Mkl1 fused to GFP (CA-Mkl1-GFP), which we have shown increases SRF/Mkl1-dependent gene expression, flipped cells into a Sun2 dominant state [31, 37, 38]. The switch induced by CA-Mkl1-GFP expression correlated with increased expression of Sun2, suggesting that LINC complex assembly in HeLa cells is controlled in part by modulating the relative levels of Sun2.

**Fig 2:**
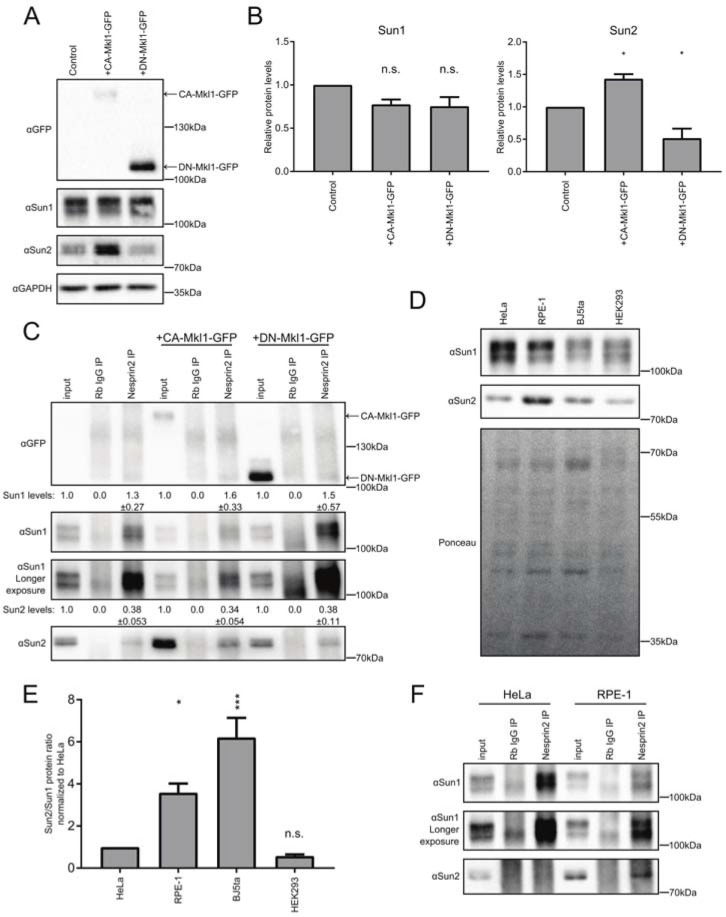
SRF/Mkl1 signaling promotes Sun2 LINC complex formation. (A) Representative blot of SUN protein levels upon SRF/MKL1 induction (CA-Mkl1-GFP) or repression (DN-Mkl1-GFP). GAPDH is a loading control. Arrows denote CA-Mkl1-GFP or DN-Mkl1-GFP. (B) Quantification of blots shown in A normalized to GAPDH. Sun2 levels correlate with SRF/Mkl1 activity (mean±SEM, n = 3). Statistical significance determined by one-way ANOVA with Dunnett’s posttest for multiple comparisons. *P < .05, n.s.-not significant. (C) Nesprin2 immunoprecipitation of SUN proteins upon activation or repression of SRF/Mkl1 pathway reveals an increase in Sun2 containing LINC complexes. Arrows denote CA-Mkl1-GFP or DN-Mkl1-GFP. Immunoprecipitation quantifications were conducted by subtracting the nonspecific Rb IgG lane and normalizing to respective input levels (mean±SEM, n = 4). (D) Representative blot of SUN protein levels across several cell types loading equal protein amounts. Ponceau included as a loading control. (E) Quantification of blots shown in D comparing relative levels of Sun2/Sun1 normalized to HeLa cells (mean±SEM, n = 3). Statistical significance determined by one-way ANOVA with Dunnett’s posttest for multiple comparisons. *P < .05, ***P < .001, n.s.-not significant. (F) Nesprin2 immunoprecipitation of SUN proteins in HeLa and RPE-1 cells. RPE-1 cells have a higher amounts of Sun2 containing LINC complexes relative to Sun1 LINC complexes.

To test this hypothesis, we measured SUN-domain protein incorporation in LINC complexes in HeLa cells expressing CA-Mkl1-GFP. As expected, CA-Mkl1-GFP expressing HeLa cells had increased Sun2 protein with no change in Sun1 protein levels (Fig 2A and 2B). We also found a notable increase in Sun2 association with Nesprin2 in HeLa cells expressing CA-Mkl1-GFP (Fig 2C). Specifically, quantification of Sun2 levels in Nesprin2 immunoprecipitations revealed that the overall efficiency of SUN protein incorporation into LINC complexes was unchanged. However, since Sun2 levels are substantially higher in cells in CA-Mkl1 expressing cells, the absolute amount of Sun2 in LINC complexes is also increased by a corresponding amount. Expression of a dominant negative form of Mkl1 (DN-Mkl1-GFP), which we have previously shown to repress SRF/Mkl1 target gene expression, led to a modest reduction in Sun2 levels but did not noticeably affect LINC complex assembly (Fig 2A and 2B) [31, 37, 38].

Due to the correlation between Sun2 protein levels and its incorporation into LINC complexes, we next asked how LINC complex composition compared among different cell types. First, we discovered that relative Sun1 and Sun2 protein levels varied across cell lines with RPE-1 and BJ5ta cells having less Sun1, and similar or higher levels of Sun2 as compared to HeLa or HEK293 cells (Fig 2D and 2E). Immunoprecipitation of Nesprin2 from RPE-1 cells revealed an increase Sun2 LINC complex abundance when compared to HeLa cells (Fig 2F). Together, these data further support the idea that the balance of Sun1 and Sun2 protein levels determines the relative abundance of LINC complexes in the nuclear envelope. SRF/Mkl1 activity in these cell lines may be one mechanism by which the cell controls relative abundance of Sun1 and Sun2.

### Sun1 has a LINC-independent role in repressing SRF/Mkl1-dependent gene activation

**Fig 3:**
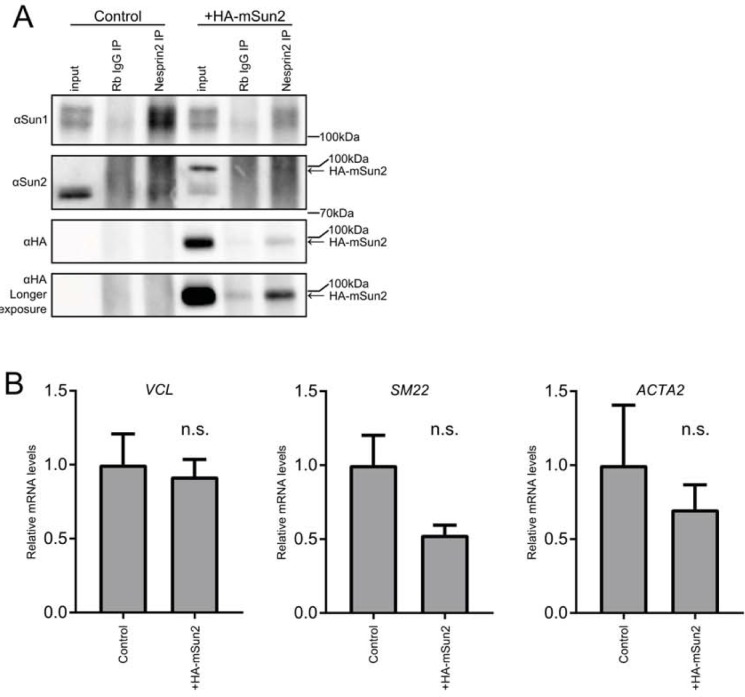
Sun2 LINCs are insufficient to induce SRF/Mkl1 signaling. (A) Nesprin2 immunoprecipitation of SUN proteins shows shifting of LINC complexes away from Sun1 upon HA-mSun2 overexpression. Arrow denotes HA-mSun2. (B) Transcript levels of SRF/Mkl1 targets upon overexpression of HA-mSun2 transgene (mean±SEM, n = 3). Statistical significance determined by ratio paired Student’s t-test. n.s.-not significant.

We next determined whether Sun2 overexpression alone was sufficient to shift the balance of LINC complexes in the nuclear envelope. Immunoprecipitation of Nesprin2 from HeLa cells that expressed mouse Sun2 (HA-mSun2) at a level ~6-fold higher than endogenous Sun2 revealed an accumulation of HA-mSun2 containing LINC complexes along with a clear decrease in the abundance of Sun1 LINC complexes (Fig 3A) [31]. These data suggest that SUN-domain proteins compete for a limiting pool of KASH-domain proteins in the nuclear envelope and that increasing Sun2 levels alters the abundance of specific LINC complex forms in the nuclear envelope.

Expression of HA-mSun2 led to a decrease in Sun1 LINC complex abundance but it did not phenocopy depletion of Sun1 in HeLa cells; while depletion of Sun1 led to an increase in SRF/Mkl1 target genes, including *VCL*, *SM22*, and *ACTA2*, HA-mSun2 expression did not as determined by quantitative PCR (qPCR) (Fig 3B) [31]. These genes have been shown to be sensitive to SRF/Mkl1 activity and are directly bound by SRF/Mkl1 through a proximal CArG box sequence [39, 40]. We therefore tested the possibility that Sun1 has a LINC-complex independent role in inhibiting SRF/Mkl1-dependent gene expression. HeLa cells that expressed human Sun1 (HA-Sun1) or a LINC defective mutant form (HA-Sun1Y775F) were generated. In addition, we created a cell line in which the entire SUN domain was removed (HA-Sun1ΔSUN), leaving only the nucleoplasmic domain and luminal coiled coils. We verified that each Sun1 mutant was defective in KASH binding using *in vitro* translated proteins (Part A in S2 Fig). Further, we confirmed that the described Sun1 proteins localized properly to the nuclear envelope and did not disrupt endogenous Sun2 localization (Part B in S2 Fig).

**Fig 4:**
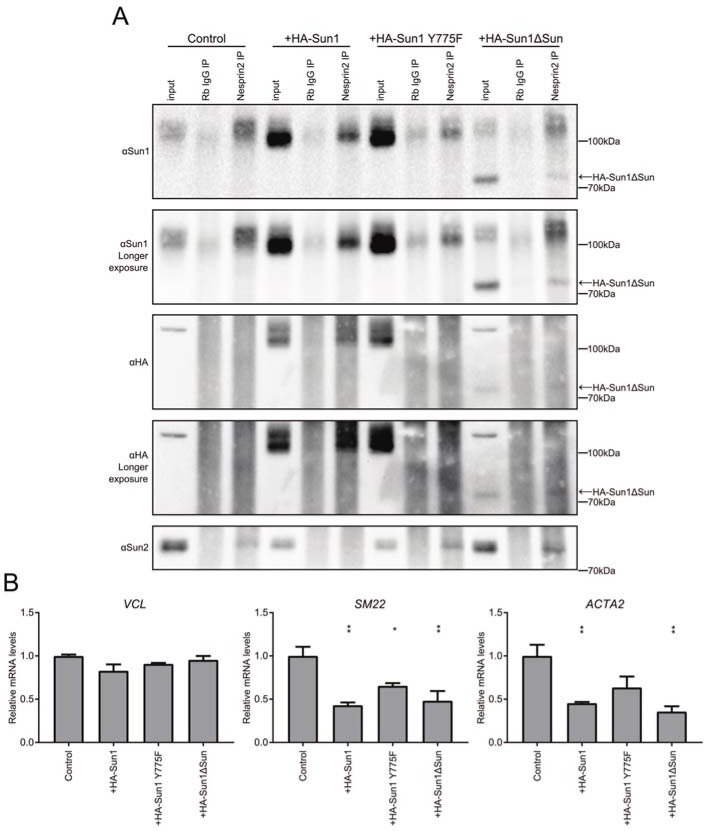
Sun1 has LINC independent roles in repressing SRF/Mkl1 activity. (A) Nesprin2 immunoprecipitation of SUN proteins upon Sun1 overexpression shows a slight increase in Sun2 binding upon Sun1 mutant but not wild type expression. Arrow denotes HA-Sun1ΔSUN. (B) Transcript levels of SRF/Mkl1 targets upon overexpression of Sun1 transgenes show a reduction in a subset of genes (mean±SEM, n = 3). Statistical significance determined by one-way ANOVA with Dunnett’s posttest for multiple comparisons. *P < .05, **P < .01.

We then interrogated LINC complex assembly in these cell lines. As expected, overexpressed HA-Sun1 was able to bind to Nesprin2 (Fig 4A), and further, it was able to displace the trace amount of Sun2 associated with Nesprin2 (Fig 4A). Both HA-Sun1Y775F and HA-Sun1ΔSUN also weakly bound to Nesprin2, possibly by forming oligomers with endogenous Sun1. Nevertheless, expression of the mutant Sun1 proteins led to an increase in the abundance of Sun2 LINC complexes. Wildtype Sun1 and KASH binding-defective Sun1 mutants have opposite effects on Sun2 LINC complex abundance.

We used qPCR to determine how wildtype and KASH binding-defective Sun1 mutants affected SRF/Mkl1-dependent gene expression. Interestingly, overexpression of both wildtype and mutant forms of Sun1 led to a reduction in the mRNA levels of the SRF/Mkl1 target genes *SM22* and *ACTA2*, but not *VCL* (Fig 4B). We cannot rule out the possibility that the remaining endogenous Sun1 was required for the observed reduction in gene expression in these experiments. Moreover, it is possible that this affect is mediated through SRF/Mkl1-independent mechanisms. Nevertheless, given that overexpression of wildtype and mutant Sun1 have different affects on Sun2 LINC complex abundance but similar affects on gene expression and the previously established role of Sun1 in inhibiting SRF/Mkl1 activity, we believe these data are most consistent with a LINC-complex independent function of Sun1 in limiting the expression of SRF/Mkl1 target genes.

Here, we have shown that Sun1 is more efficiently incorporated into LINC complexes in HeLa cells under normal growing conditions. The disparity may be caused by differences in affinity stemming from Sun2 coiled coil-based regulation or other unidentified factors [41]. This balance is cell type specific and is likely regulated in part by SRF/Mkl1 signaling, which selectively promotes Sun2 levels to increase its LINC incorporation. From our previous work, Sun2 LINC complexes have been shown to be necessary for promotion of RhoA and SRF/Mkl1-dependent gene expression [31]. The biochemical data presented here suggest one mechanism underlying this inhibition the sequestration by Sun1 of Nesprin proteins away from Sun2. Interestingly, we have additionally found that Sun1 likely has a LINC complex-independent role in limiting SRF/Mkl1-dependent gene expression. Although the mechanism of SRF/Mkl1 inhibition is not known, an intriguing possibility is that the nucleoplasmic region of Sun1 restricts the ability of Lamin A/C and/or Emerin to promote SRF/Mkl1-dependent transcription [10, 13, 42, 43]. The emerging picture is one in which Sun1 opposes Sun2 LINC complex function through multiple mechanisms.

## Materials and methods

### Cell culture

HeLa stable cell lines were established and cultured as previously described (Thakar *et al*, 2016). Expression of transgenes in HeLa stable cells lines was induced by addition of doxycycline (1ug/ml final concentration) for 24-72h. RPE-1 cells were cultured in DMEM/F12 (1:1) with 10% Fetal Bovine Serum (FBS; Sigma-Aldrich), .01mg/ml hygromycin B and penicillin/streptomycin (P/S; ThermoFischer). BJ5ta cells were cultured in DMEM/Medium 199 (4:1) with 10% FBS, .01mg/ml hygromycin B and P/S. HEK293 cells were cultured in DMEM with 10% FBS, and P/S. All cell lines were kept at 37°C with 5% CO_2_. siRNA knockdown was as previously described [31].

### GST-KASH bead purification

GST-KASH, containing the last 29 amino acids of Nesprin2, and GST-KASH^mut^, were expressed in *E*. *coli* using pGEX-6P. Cells were resuspended in 20ml buffer (50mM Tris pH 7.4, 150mM NaCl, 1mM EDTA, 1mM PMSF) per liter of initial culture. After lysis by cell disruptor, Triton X-100 was added to a final concentration of .5%. Lysate was clarified by 40min 15000rpm spin 4°C. 1ml of glutathione agarose beads were added per liter of initial culture and incubated for 3-4h 4°C. Beads were washed (50mM Tris pH 7.4, 150mM NaCl, 1mM EDTA, .1% Triton X-100) four times before being resuspended at 1mg/ml.

### GST-KASH pulldown

Cell were washed in cold PBS, lysed in RIPA buffer (50mM Tris pH 8.0, 150mM NaCl, 1% Triton X-100, .1% SDS, .1% sodium deoxycholate) with Protease Inhibitor (PI; Roche) and Benzonase (1:1000 dilution; Sigma-Aldrich) then collected by scraping. 20min post-lysis, cells were centrifuged for 10min 10000rpm at 4°C to remove aggregates. 100μg of protein determined by Bradford was added to 50ug of GST-KASH beads diluted in RIPA buffer to 200μl. After 1h 4°C with gentle agitation, beads were washed four times in GST-KASH Wash buffer (GW; 50mM Tris pH 7.4, 150mM NaCl, .1% Triton X-100) and protein was eluted with SDS-PAGE loading buffer. Inputs represent 5% of total. For *in vitro* translation experiments, 10μl TNT SP6 Quick Coupled Transcription/Translation System (Promega) was mixed with .5μl 1mM Methionine, 1.5μl H_2_O and .5μl DNA and incubated for 90min 30°C. Protein was diluted in RIPA and spun at for 10min 10000rpm 4°C to remove aggregates prior to addition to beads as previously described.

### GFP-Trap LINC complex off-rate experiments

Cells were harvested as described in GST-KASH pulldown experiments and clarified lysates were incubated with GFPTrap beads (ChromoTek) for 1h 4°C. After four washes in GW, protein was eluted with SDS-PAGE loading buffer. For the off-rate experiments, the beads were instead diluted in 1ml RIPA buffer containing 5μM GST-KASH post washing for the specified times before washing in GW four times and eluting in SDS-PAGE loading buffer.

### Nuclear isolation and immunoprecipitations

Cells were washed in cold PBS and collected by scraping in hypotonic lysis buffer (10mM HEPES pH 7.9, 10mM KCl, 1.5mM MgCl_2_) supplemented with PI. After 15min on ice, 10% NP-40 (.5% final concentration) was added. Cells were vortexed for 10sec before collecting nuclei by a 5min 1000rpm spin 4°C. Nuclei were resuspended in RIPA buffer with PI and Benzonase before a 10min 10000rpm spin to remove aggregates. 2μg antibody was added to the lysate for 1h 4°C before mixing with magnetic protein A/G beads (ThermoFischer) for 4h 4°C. Beads were washed in GW and eluted as described for the GST-KASH pulldowns. Inputs represent 5% of total.

### Preparation of nonreduced and fixed samples

Cells were washed into PBS or PBS containing .5% formaldehyde for 10min room temperature. Fixation was stopped by 50μl of 1.375M glycine before two washes in PBS and addition of RIPA with PI and Benzonase. All samples were mixed with SDS-PAGE loading buffer except nonreduced samples which were mixed with SDS-PAGE loading buffer lacking β-mercaptoethanol (βME).

### Immunofluorescence

Cells were grown on glass coverslips and fixed with ice cold methanol for 10min at -20⁰C. Blocking Buffer (BB; TBST + .1% Triton X-100 + 20mg/ml BSA + .02% Sodium Azide) was added for 30min. Primary antibodies listed in S1 Table were then added for 45min. Cells were washed three times 5min each in BB before addition of secondary antibodies for 30min. Cells were then washed in BB before staining with Hoescht (2μg/ml in BB). After two washes in BB and then in PBS, coverslips were mounted with ProLong Gold antifade (Molecular Probes). Images were taken with a Leica SP5 laser scanning confocal microscope with a 63X oil immersion objective.

### Protein and RNA methods

Cells were lysed in SDS-PAGE loading buffer. Primary antibodies are listed in S1 Table. HRP coupled goat anti-mouse or–rabbit antibodies were added at the same dilution as primary antibody. RNA isolation, reverse transcription and qPCR was conducted as described previously except 100ng of RNA was used for cDNA synthesis (Thakar *et al*, 2006). *ACTA2* primers: 5’TATCCCCGGGACTAAGA and 5’CCTTACAGAGCCCAGAG.

## Supporting information

S1 Fig Sun protein luminal domains bind GST-KASH. (A) Representative blot of Sun1 and Sun2 knockdown by siRNA to demonstrate specificity of αSun1 and αSun2 antibodies used in this study. (B) Pulldown of endogenous SUN proteins with GST-KASH on beads is reduced upon GFP-SR-KASH overexpression. The dashed line represents an omitted lane. (C) Representative blot of *in vitro* translated HA-tagged SUN protein luminal domains binding GST-KASH on beads. GST-KASH^mut^ lacks residues critical for SUN protein interaction and does not exhibit notable binding.
(PDF)

S2 Fig LINC defective Sun1 and Sun2 mutant proteins localize to the nuclear envelope. (A) Representative blot of *in vitro* translated HA-tagged Sun1 and Sun2 luminal domains with the indicated mutations. Luminal Sun1ΔSUN consists of amino acids 309–621. (B) Cell lines expressing HA-tagged Sun1 constructs localize at the nucleus without disrupting endogenous Sun2. Images are max intensity z-projections. In the merge image, DNA is pseudo-colored in blue, induced HA-tag protein in green and the indicated endogenous Sun protein in red. Scale bar is 10μm.
(PDF)

S1 Table Primary antibodies used in this study. (DOCX)

## Abbreviations

LINC: LInkers of Nucleoskeleton and Cytoskeleton
INM: inner nuclear membrane
KASH: Klarsicht, ANC-1, and Syne homology
SUN: Sad1, UNC84
