# Supplementary figures and images for: Differential incorporation of SUN-domain proteins into LINC complexes is coupled to gene expression
Source: PLoS One. 2018 May 29;13(5):e0197621. doi: 10.1371/journal.pone.0197621 (PMC5973619; doi:10.1371/journal.pone.0197621)

Figure S1

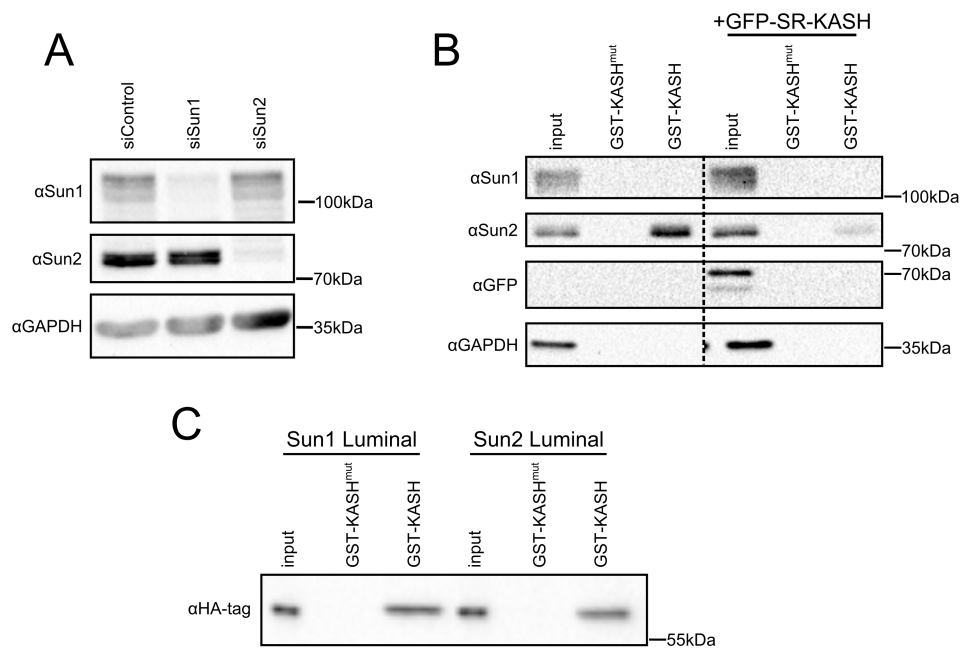

Supplement: S1 Fig — (A) Representative blot of Sun1 and Sun2 knockdown by siRNA to demonstrate specificity of αSun1 and αSun2 antibodies used in this study. (B) Pulldown of endogenous SUN proteins with GST-KASH on beads is reduced upon GFP-SR-KASH overexpression. The dashed line represents an omitted lane. (C) Representative blot of in vitro translated HA-tagged SUN protein luminal domains binding GST-KASH on beads. GST-KASHmut lacks residues critical for SUN protein interaction and does not exhibit notable binding. (PDF) [file pone.0197621.s001.pdf]

Figure S2

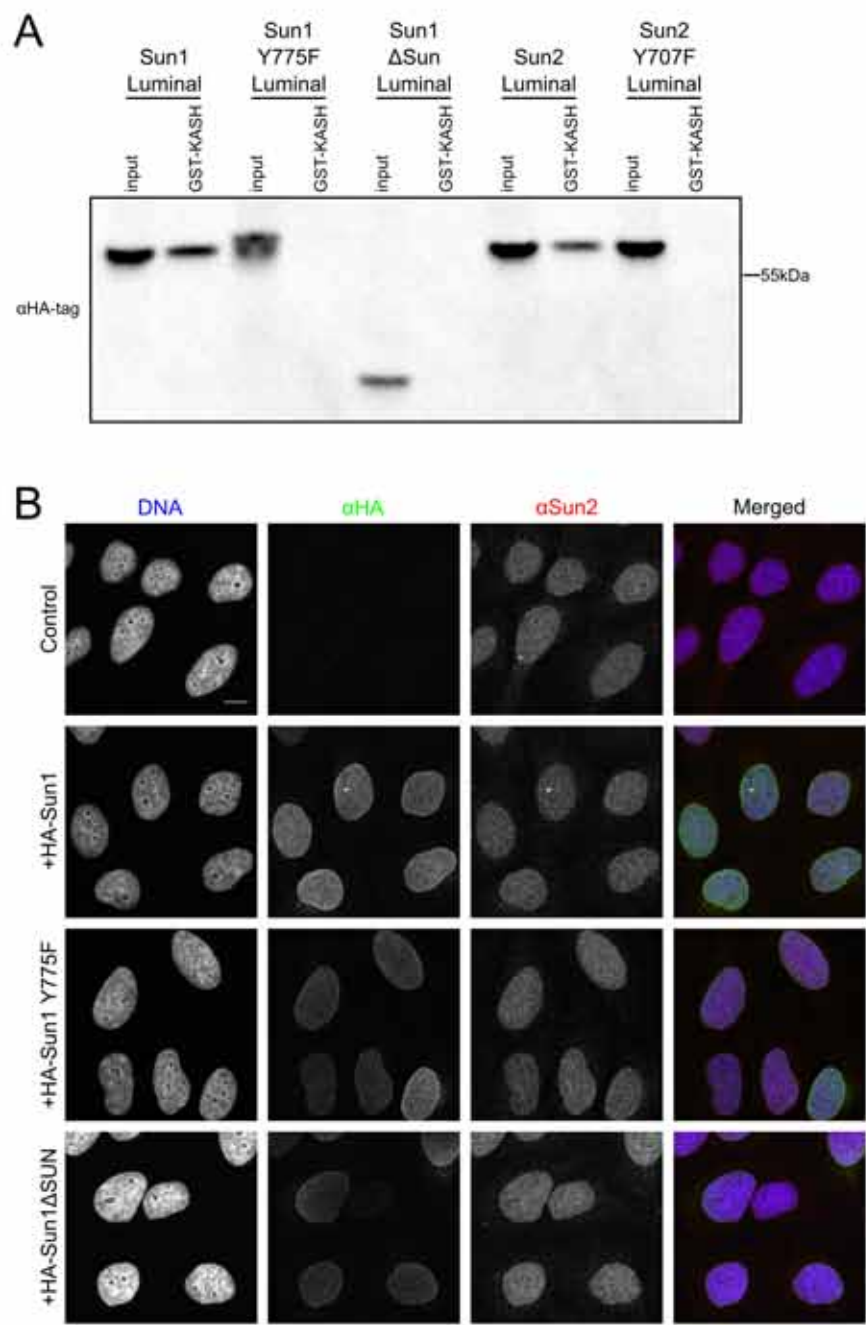

Supplement: S2 Fig — (A) Representative blot of in vitro translated HA-tagged Sun1 and Sun2 luminal domains with the indicated mutations. Luminal Sun1ΔSUN consists of amino acids 309–621. (B) Cell lines expressing HA-tagged Sun1 constructs localize at the nucleus without disrupting endogenous Sun2. Images are max intensity z-projections. In the merge image, DNA is pseudo-colored in blue, induced HA-tag protein in green and the indicated endogenous Sun protein in red. Scale bar is 10μm. (PDF) [file pone.0197621.s002.pdf]
