# Supplementary material for: Differential incorporation of SUN-domain proteins into LINC complexes is coupled to gene expression
Source: PLoS One. 2018 May 29;13(5):e0197621. doi: 10.1371/journal.pone.0197621 (PMC5973619; doi:10.1371/journal.pone.0197621)
Supplement: S1 Table — (DOCX) [file pone.0197621.s003.docx]

| **Identifier** | **Provider** | **Catalog No.** | **Usage** |
| --- | --- | --- | --- |
| Rabbit αSun1 | Abcam | ab124770 | 1:1000 WB |
| Rabbit αSun2 | Carroll lab | - | 1:1000 WB |
| Rabbit αSun2 | Abcam | ab124916 | 1:1000 WB, 1:200 IF |
| Rabbit αGFP | Santa Cruz Biotechnology | sc-8334 | 1:1000 WB |
| Rabbit αNesprin1 | Biolegend | 902701 | 2μg IP |
| Rabbit αNesprin2 | Santa Cruz Biotechnology | sc-99181 | 2μg IP |
| Rabbit αGAPDH | Cell Signaling Technology | 2118 | 1:4000 WB |
| Rabbit αSM22 | Abcam | ab14106 | 1:2000 WB |
| Mouse αVinculin | Sigma | B4505 | 1:1000 WB |
| Rabbit αHA | Carroll lab | - | 1:1000 WB |
| Mouse αHA | Biolegend | 901513 | 1:200 IF |

S1 Table
